# Comparative Study of Carbon Nanotube Composites as Capacitive and Piezoresistive Pressure Sensors under Varying Conditions

**DOI:** 10.3390/ma15217637

**Published:** 2022-10-30

**Authors:** Jihyeon Oh, Dong-Young Kim, Hyunwoo Kim, Oh-Nyoung Hur, Sung-Hoon Park

**Affiliations:** Department of Mechanical Engineering, Soongsil University, 369 Sangdo-ro, Dongjak-gu, Seoul 06978, Korea

**Keywords:** carbon nanotube, piezoresistive effect, capacitive effect, pressure sensor, polymer composite

## Abstract

Conducting polymer composites consisting of carbon nanotubes (CNTs) as a conductive filler and polydimethylsiloxane (PDMS) as a polymer matrix were fabricated to investigate their capacitive and piezoresistive effects as pressure sensors. The pressure-sensing behavior and mechanism of the composites were compared in terms of basic configuration with a parallel plate structure. Various sensing experiments, such as sensitivity, repeatability, hysteresis, and temperature dependence according to the working principle, were conducted with varying filler contents. The hysteresis and repeatability of the pressure-sensing properties were investigated using cyclic tensile tests. In addition, a temperature test was performed at selected temperatures to monitor the change in the resistance/capacitance.

## 1. Introduction

Flexible pressure sensors attract considerable research efforts because of their significant application potential in the fields of electronic skin [1,2,3,4], robotics [5,6,7], and wearables [8,9]. For example, robots and other machines gain sensory capabilities when equipped with electronic skin or flexible pressure sensors that act in the same way as human skin. Therefore, the fabrication of flexible pressure sensors with high performance and flexibility has become an important research topic. However, obtaining high sensitivity, flexible pressure sensors with low cost and convenience is still challenging. In particular, the absence of a high-performance pressure sensor is a significant obstacle. Most pressure sensors commercially available today are used only in the high-pressure range or are limited in several aspects, such as flexibility and sensitivity. On the other hand, devices such as robots and wearables must provide accurate, sensitive, and reliable performance at a low pressure. Therefore, more research is needed on the development of sensitive sensors.

Various studies have been conducted on conductive polymer composites for fabricating flexible pressure sensors [10,11]. Recently, studies have been conducted on manufacturing composites using carbon nanomaterials as conductive fillers [12]. Flexible pressure sensors can be fabricated using conductive fillers such as metallic nanomaterials, graphene, graphene oxide, and carbon nanotubes (CNTs). In particular, CNTs have been widely used in pressure sensors thanks to their unique electrical and mechanical properties. For example, the high aspect ratio of carbon nanotubes readily forms conductive networks, showing measurable changes in electrical resistance and capacitance. PDMS is also a commonly used elastomer. Therefore, these materials were chosen for the fabrication of flexible composites.

According to the sensing principle, flexible pressure sensors can be classified into piezoelectric [13], triboelectric [14,15], piezoresistive [16,17], and capacitive [18,19] sensors. These sensors have different advantages depending on the detection mechanism. For example, piezoelectric pressure sensors are based on the piezoelectric effect, which has the advantages of high sensitivity and fast response, but cannot effectively measure static pressure [20]. Triboelectric pressure sensors do not have an external power supply because they use contact charging to generate a voltage signal in response to physical contact. However, triboelectricity is affected by many factors, including humidity, temperature, pressure, and the velocity of the applied force. The main parameter of a piezoresistive pressure sensor is its rate of change in resistance. The change in resistance is usually caused by internal or contact resistance changes due to structural changes in sensitive materials. Although piezoresistive sensors have the advantages of accessible data collection and a simple structure, the sensing material is easily affected by environmental factors [21]. In addition, capacitive pressure sensors are widely used owing to their low power consumption, fast response speed, simple structure, and ability to optimize sensor performance according to voltage and frequency [22]. Thus, piezoresistive and capacitive types, which have simple structures and exhibit efficient data collection, are generally used. Therefore, the pressure-sensing behavior based on the two mechanisms was studied using the basic parallel-plate structure under various conditions in the pressure field. Moreover, a performance comparison study was performed on the conductive filler content based on the working principle that helped to understand the pressure sensing mechanism.

In this study, carbon nanotubes (CNTs), a conductive carbon nanofiller, and polydimethylsiloxane (PDMS), a polymer matrix, were used to fabricate a flexible pressure sensor, and its piezoresistive and capacitive pressure-sensing characteristics were compared. A three-roll milling method was used to manufacture the CNT/PDMS and the filler was evenly dispersed. First, we analyzed the dispersion and percolation behaviors. We then measured the sensitivity of both pressure sensors and recorded the changes in resistance and capacitance at a low pressure to investigate the differences in repeatability and hysteresis. Finally, the tendency and detection mechanism were investigated using a pressure test to confirm the temperature dependence of the two pressure sensors.

## 2. Materials and Methods

### 2.1. Materials

Functionalized CNTs improve mechanical properties in some reported cases [23]. However, some functionalizations destroy the CNT shell and reduce the conductivity of the composites, making it difficult to obtain consistent results [24]. Therefore, in this paper, non-functionalized CNTs were used. The employed CNTs were purchased from KB-Element (Gyeonggi-do, Korea) and featured an outer diameter of 5 nm, a bundle length in the range of 10–20 μm, and a true density of 1.4 g/m^3^. Polydimethylsiloxane (PDMS; Sylgard 184, Dow Corning, Midland, MI, USA) was used as the base polymer matrix.

### 2.2. Preparation of CNT/PDMS Composites and Fabrication of the Pressure Sensor

CNT/PDMS composites were fabricated with different CNT contents. A paste mixer (Daehwa, Seoul, Korea) and a three-roll milling machine (Intech, Gyeonggi-do, Korea) were used to disperse the CNTs in the PDMS. First, PDMS was prepared with a premixing base agent (A) and curing agent (B) (mass ratio A/B = 10:1), and then CNTs were added to PDMS. Next, using a paste mixer, it was mixed at 500 rotations per minute (rpm) for 30 s, and then the paste was mixed continuously at 1500 rpm for 60 s. Subsequently, three-roll milling was carried out for 5 min to ensure uniform dispersion [25,26]. Finally, the mixture was compressed and cured at 170 °C for 1 h 30 min and under 15 MPa using a hot press (Qmesys Inc., Uiwang-si, Gyeonggi-do, Korea) to obtain a 500 μm thick flat film. Thus, the obtained CNT/PDMS composites were used in the fabrication process of the pressure sensor. For the flexible performance of this composite, see previous studies [27,28].

To compare the pressure sensing capabilities of the piezoresistive and capacitive types in the basic parallel plate configuration, the pressure sensor in this study had a flat three-layer sandwich structure. First, two electrode layers were fabricated by attaching copper tape to the glass substrate. Then, a sensing layer with a size of 1.5 × 1.5 cm^2^ composed of a CNT/PDMS composite was disposed of between them. Finally, the contact resistance generated for each layer was reduced to precisely measure the electrical properties [14,22,23]. Therefore, after the electrodes and composite were sequentially stacked, a weight of 60 g was placed thereon and the assembly was encapsulated with tape to manufacture a pressure sensor.

### 2.3. Device Characterization and Test Conditions

To confirm that the CNTs of the prepared composite were well dispersed in PDMS, the sample was fractured using liquid nitrogen and the shape and dispersion were observed by SEM (Gemini SEM 300, ZEISS Inc., Land Baden-Württemberg, Germany). The equipment was operated at an acceleration voltage of 5 kV.

Composites of varying contents were prepared to measure the electrical properties and percolation threshold of the composites. Three samples (5 × 5 × 0.5 mm^3^) were obtained from the composite corresponding to each content and the average values of the electrical properties were measured. First, the samples were etched by ultraviolet (UV) rays for 300 s using a UV ozone room (JSE Co., Seoul, Korea) to enhance the electrical contact between the samples and the silver paste (Protavic, Levallois-Perret, France). Then, the silver paste was used to cover both ends of the surface of the samples as electrodes. Next, it was cured at 120 °C for 1 h. Finally, the resistance was measured using a two-point probe method with a Keithley DMM 7510 multimeter (Keithley, Cleveland, OH, USA).

A parallel plate-structured sensor was fabricated using various composites and their dielectric properties were measured. First, a sample with dimensions of 15 × 15 × 0.5 mm^3^ was prepared. Pt was then coated on the upper and lower surfaces of the sample using a Quorum Q150R S sputter coater (Quorum Technologies Ltd., Lewes, UK) to fabricate an electrode. Next, the sensor used a GW INSTEK LCR-6300 m (GWINSTEK, New Taipei, Taiwan) to set the driving voltage to 1 V and the driving frequency to 0.1 MHz and measured the capacitance and dielectric loss tangent.

To measure the pressure-sensing behavior of the composites, a custom press machine with strain gauges (NAMIL, Incheon, Korea) was used to apply pressure while simultaneously measuring the resistance and capacitance with an LCR meter. First, the load cell compressed the sensor in the pressure range of 0–200 kPa at a speed of 1 mm/min. In addition, the LCR meter operation mode was measured as C_P_-R_P_, with a frequency of 100 kHz and voltage of 1 V. Next, the load–unloading experiment was performed with a delay of 30 s for each process for 3000 s in a pressure range of 0–50 kPa. Finally, hysteresis was measured through one cycle of load–unloading experiments in a pressure range of 0–50 kPa.

To measure the change in the pressure-sensing behavior of the composites according to the temperature, a hot plate was manufactured using a customized CNT film. Two CNT films with dimensions of 25 × 76 mm^2^ were prepared. An electrode was made by attaching copper tape to both ends of the CNT film using silver paste. Before attaching the electrodes, the samples were UV-etched for 300 s in a UV ozone chamber to strengthen the electrical contact between the CNT film and silver paste. After connecting the electrodes of the two manufactured CNT films to a power supply, a voltage was applied to maintain them at a constant temperature. Then, the composite to be measured was placed between the two CNT film hot plates and pressure was applied at a constant speed with a customized press machine to measure the pressure-sensing behavior. The temperature was monitored using a Keithley DMM 2110 multimeter (Keithley, Cleveland, OH, USA) with a thermometer connected to the composite to ensure that a constant temperature was maintained.

## 3. Results and Discussion

### 3.1. Morphology Analysis

**Figure 1:**
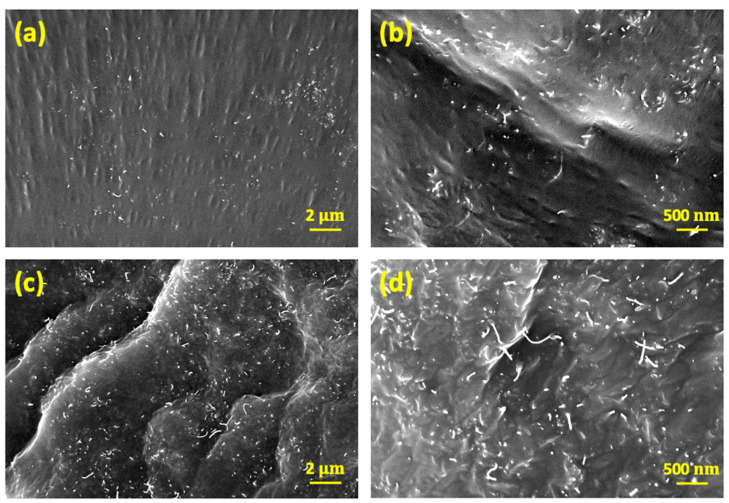
SEM images of composites (**a**) CNT/PDMS 1 wt% at low resolution, (**b**) CNT/PDMS 1 wt% at high resolution, (**c**) CNT/PDMS 5 wt% at low resolution, and (**d**) CNT/PDMS 5 wt% at high resolution.

Figure 1 shows the SEM images of the fractured surface of the low- and high-content carbon nanotube composites (CNT/PDMS). Figure 1a,b show cross-sectional SEM images of the CNT/PDMS composite with a low CNT content of 1 wt%. The figures correspond to low- and high-resolution images, respectively. Figure 1c,d are low and high-resolution cross-sectional SEM images, respectively, of a CNT/PDMS composite with a high CNT content of 5 wt%. The SEM images show the differences according to the dispersion and content of the nanofillers. It can be confirmed that, regardless of their content, all fillers are uniformly dispersed in the polymer matrix, owing to three-roll milling.

### 3.2. Electrical Conductivity and Percolation Threshold

**Figure 2:**
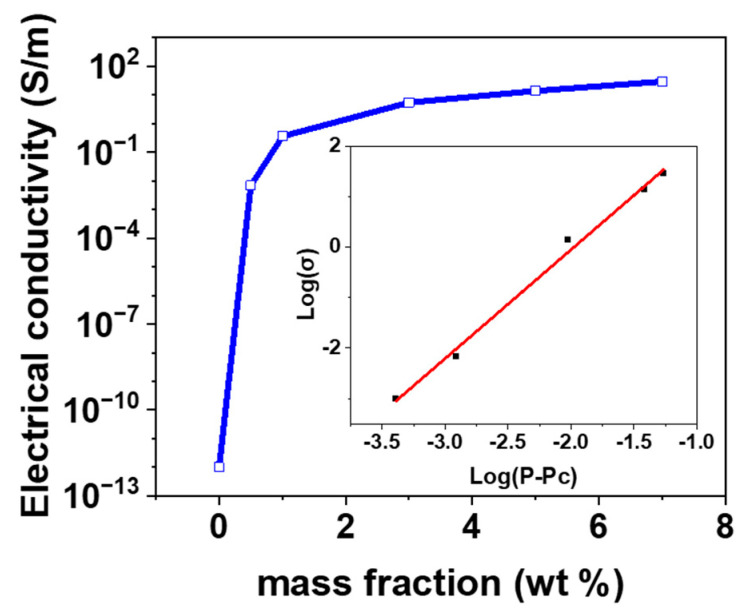
The electrical conductivity of CNT/PDMS composites as a function of mass fraction (wt%).

The electrical conductivity and percolation threshold were investigated to confirm the electrical properties of the CNT/PDMS composites in a pressure field. Figure 2 shows the electrical conductivity and percolation threshold according to the filler content of the CNT/PDMS composites. Furthermore, the data of 0.4, 1, 3, 5, and 7 wt% are shown in order. Here, 0.4 wt%, where the initial conductivity was taken, was indicated to find the percolation threshold. In addition, the electrical conductivity was saturated from 5 wt%. Therefore, data exceeding 7 wt% were not displayed. The electrical conductivity of the composite was calculated using Equation (1).

(1) $$\sigma_{conductivity}=\frac{l}{RA}$$

where $\sigma_{conductivity}$ is the electrical conductivity, 𝑅 is the resistance, 𝐴 is the cross-sectional area of the sample, and 𝑙 is the distance between the two electrodes. Because CNTs have a large aspect ratio, they can rapidly form electrical networks [29]. Therefore, a sharp increase in electrical conductivity is observed in CNT/PDMS at low content values, as shown in Figure 2. This situation is called percolation, characterized by a sharp drop of several orders of magnitude in resistivity [30].

The electrical results of the composites can be explained by percolation theory. Percolation theory predicts that the electrical conductivity of polymer composites increases significantly at the critical volume fraction of CNTs. This is believed to be due to two conduction mechanisms [31,32]:

(1) Electron hopping (or quantum tunneling) at the nanoscale;
(2) Conductive networks at the microscale.

Therefore, the insulating polymer becomes electrically conductive when the filler content exceeds a certain threshold. Therefore, the relationship between the conductivity of the composites and the filler content is defined by the percolation theory as follows:

(2) $$\sigma_{c}\propto \sigma_{0}{\left(p-p_{c}\right)}^{t}$$

where $\sigma_{c}$ is the electrical conductivity, *σ_0_* is the reference electrical conductivity, *p* is the CNT content, *p_c_* is the electrical percolation threshold, and t is a critical index. In this study, for CNT/PDMS, *p_c_* and *t* are calculated using Equation (2) to be 0.35 wt% and 2.15, respectively. The obtained results (*p_c_* and *t*) followed the same trend as in previous studies [27,33,34,35].

### 3.3. Dielectric Properties and Dielectric Loss

**Figure 3:**
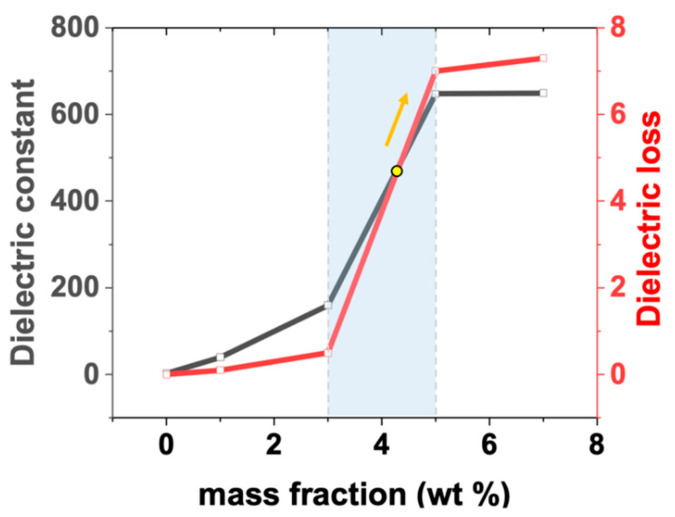
The dielectric constant and dielectric loss of CNT/PDMS composites as a function of mass fraction (wt%). The arrow indicates the reversed section.

Adding CNTs to the PDMS matrix can improve the dielectric properties of the material. The dielectric constant of the CNT/PDMS composite is defined as

(3) $$\varepsilon_{r}=\frac{Cd}{\varepsilon_{0}A}$$

where *ε_r_* is the dielectric constant of the composite, *ε*_0_ is the dielectric constant of vacuum, *C* is the capacitance, *d* is the distance between the two electrodes, and A is the cross-sectional area of the electrode. C and the dielectric loss tangent D were measured using an LCR meter (0.1 MHz). *ε_r_* and dielectric loss related to the CNT mass fraction of the CNT/PDMS composites are shown in Figure 3. Figure 3 shows data for pure PDMS and CNT/PDMS (1, 3, and 5 wt%). *ε_r_* and dielectric loss tended to increase with an increase in CNT mass fraction. *ε_r_* shows an apparent increase when the CNT mass fraction reached a particular value (5 wt%). When the mass fraction of CNTs increased from 3 to 5 wt%, *ε_r_* increased by approximately fourfold from 160 to 650. The *ε_r_* curve shows that it is highly dependent on the mass fraction. When the interface between CNTs and PDMS increases in proportion to the mass fraction of CNTs, the electron mobility and interfacial polarization can be further improved, leading to an improved dielectric constant [36]. In addition, the relatively high aspect ratio of CNTs contributes to a faster increase in *ε_r_* than that by conductive fillers with a relatively low aspect ratio [35]. A power–law equation can explain this phenomenon based on percolation theory [36,37].

(4) $$\varepsilon_{r}\propto \varepsilon_{r\;PDMS}{\left(f_{c}-f_{CNT}\right)}^{-S},\;f_{c}\geq f_{CNT}$$

where *ε_r_ _PDMS_* is the dielectric constant of PDMS, *f_c_* is the percolation threshold, *f_CNT_* is the filler content, and *S* is the critical index. According to Equation (4), *ε_r_* is directly proportional to the CNT filler mass fraction (*f_CNT_*). Therefore, the rate of increase in *ε_r_* is accelerated when the *f_CNT_* approaches *f_c_* of the CNT/PDMS composite. The trend of the mass-dependent dielectric constant curve of the CNT/PDMS composite in Figure 3 agrees with the percolation theory.

Figure 3 shows that the D increased as the CNT mass fraction increased. The loss tangent represents the leakage current during charge storage. The greater the loss tangent, the greater the leakage current during charge storage, which has an adverse effect on charge storage. Therefore, C is affected by dielectric loss. When the CNT mass fraction is 3 wt% or less, the rate of increase in *ε_r_* was higher than that in D. However, this was reversed when the CNT mass fraction increased to 5 wt%. Therefore, as the CNT mass fraction increases, the rate of increase of D becomes faster than that of *ε_r_*, thus the output performance deteriorates. Similarly, this phenomenon can be explained by the percolation theory. According to the percolation theory, when the concentration of conductive fillers is close to the percolation threshold, the composite often exhibits a transition of electrical and dielectric properties because of the 3D conductive path of the newly formed composite [38,39]. Therefore, for samples approaching *f_c_*, *tanδ* is mainly caused by conduction loss and polarization loss of space charge, of which conduction loss contributes much more to the output performance [40], thus reducing the output performance.

### 3.4. Pressure Sensing Properties

#### 3.4.1. Sensitivity

The experiment was performed using a customized press machine for CNT/PDMS composites to compare the changes in properties and sensitivity depending on the working principles. For the safe operation of the load cell, the maximum load was limited to 200 kPa. Therefore, it was impossible to characterize the pressure-sensing range for loads greater than 200 kPa. The sensitivity was calculated using Equations (5) and (6) in the initial linear range (~25 kPa).

S = (∆*R*/*R*_0_)/∆*P* (5)

where S is the sensitivity, ∆*R* is the change in the resistance, *R*_0_ is the initial resistance when no pressure is applied, and ∆*P* is the applied pressure.

**Figure 4:**
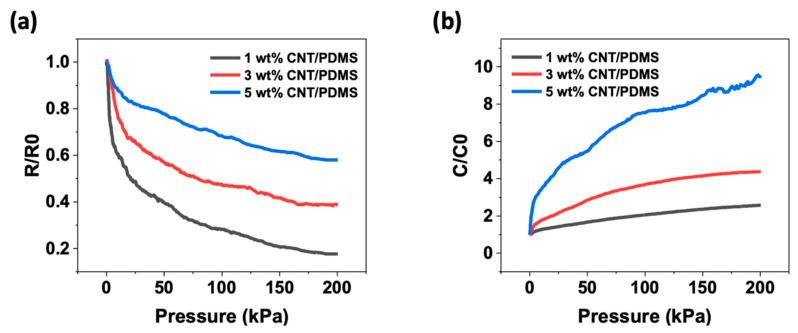
Normalized change in (**a**) resistance (*R*/*R*_0_) and (**b**) capacitance (*C*/*C*_0_) versus pressure with varying contents of CNTs in the composites.

S = (∆*C*/*C*_0_)/∆*P* (6)

where S is the sensitivity, ∆*C* is the change in the capacitance, *C*_0_ is the initial capacitance when no pressure is applied, and ∆*P* is the same as that in Equation (5). Figure 4 shows the change in relative resistance (*R*/*R*_0_) (Figure 4a) and relative capacitance (*C*/*C*_0_) (Figure 4b) as a function of pressure for various CNT mass fractions.

As shown in Figure 4a, *R/R*_0_ of the CNT/PDMS composites decreases when pressure is applied, which can be explained by the piezoresistive effect. The change in the resistance of the composite was caused by a change in the conductive network composed of CNTs. The external pressure brings the CNTs closer to each other. When the spacing between the CNTs is sufficiently small, a tunneling effect occurs, forming a local conductive path [41,42,43]. When a local conductive pathway penetrates the insulating matrix, an effective conductive pathway (ECP) is formed, contributing to the conductivity of the composite. Compression alters ECP, thereby altering the resistance of the composite. Thus, ECP may break or form during compression. The formation (destruction) of ECPs caused an increase (decrease) in the number of ECPs and contributed to a decrease (increase) in the resistance of the composite. Furthermore, the lower the CNT content, the higher the pressure sensitivity. In this experiment, when the CNT content was 1 wt%, the resistance change rate was the highest. As a one-dimensional material, CNTs can easily connect and form a stable electrical path, even at low concentrations. However, when the CNT content is high, it is difficult to create new electrical pathways owing to the sufficient contact. As a result, the pressure sensitivity is lower than that when the CNT content is low [33]. Therefore, in this study, the composite with a CNT content of 1 wt% had the highest sensitivity, and the composite with a CNT content of 5 wt% had the lowest sensitivity for resistive pressure sensors. The sensitivities of the linear range of the composites according to increasing content were 0.083 kPa^−1^, 0.062 kPa^−1^, and 0.05 kPa^−1^, respectively.

As shown in Figure 4b, the change in (*C*/*C*_0_) of the CNT/PDMS composites increased under pressure. This can be explained by two factors. The first was compression upon application of pressure to the composite. The dielectric constant of the compressed composite increased by increasing the concentration of CNTs in the vertical direction. Second, an electric field between the two electrodes increases the capacitance because the distance between the electrodes decreases as a composite is compressed. Further, the interfacial polarization between the CNT filler and PDMS matrix was also enhanced, increasing the capacitance [44]. As shown in Figure 4b, the pressure sensitivity and signal magnitude increase with an increase in the CNT content. In this study, when the CNT content was 1 wt%, the capacitance change rate was the lowest. This is because the concentration of the filler in the compressed composite is relatively small when pressure is applied compared with the high concentration of CNTs. Therefore, the sensitivity and signal magnitude were best when the CNT content of CNTs was 5 wt%. However, as shown in Figure 3, when the content was 5 wt%, the dielectric loss was greater than the rate of increase in the dielectric constant. Thus, its power performance deteriorated, making it unsuitable as a capacitive pressure sensor. Therefore, based on the results of this study, when the CNT mass fraction was 3 wt%, the signal was stable and the sensitivity and signal magnitude were relatively large, making it more suitable as a capacitive pressure sensor than 5 wt%. The sensitivities of the linear section of the composites according to increasing content were 0.056 kPa^−1^, 0.086 kPa^−1^, and 0.184 kPa^−1^, respectively.

#### 3.4.2. Cyclic Test and Hysteresis

An ideal pressure sensor should provide a fast linear response, reproducibility, and reliability [45]. However, in polymer-based pressure sensors with viscoelastic behavior, hysteresis is observed, in which the conductivity does not return to its initial state even when the external force is removed [46,47]. Consequently, hysteresis causes instability under pressure, resulting in different loading and unloading response curves. However, the amplitude of this phenomenon can be reduced by rearranging the inner filler of the composite through an initial repeated pressure cycle [48]. In this study, repeatability and hysteresis were investigated through a cyclic test to test the efficiency of the pressure sensor according to the working principle.

**Figure 5:**
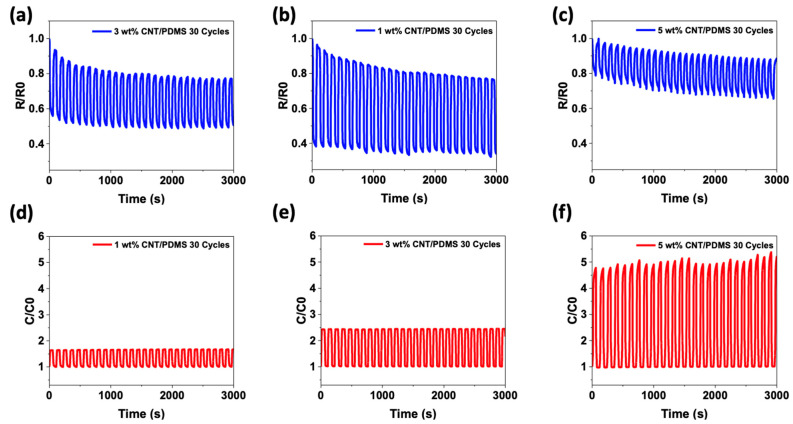
Normalized change in (**a**–**c**) resistance (R/R_0_) and (**d**–**f**) capacitance (C/C_0_) under cyclic pressure deformation at 50 kPa with varying contents of CNTs in the composites: (**a**,**d**) 1 wt%, (**b**,**e**) 3 wt%, and (**c**,**f**) 5 wt% CNT/PDMS.

The repeatability determines whether the sensor can operate and remains stable for a long time. Therefore, repeated load–unloading tests were conducted at 50 kPa after five pre-pressing cycles to confirm the repeatability of the sensor through a continuous pressure cycle. Figure 5 shows the relative resistance and capacitance change of the load–unloading cycle test for the CNT/PDMS composites for 3000 s. The results indicated that the composites achieved good resilience and reproducibility in repeated load–unloading cycles for both properties. However, the capacitive-type pressure sensor exhibited better repeatability. In the case of Figure 5e, stable repeatability was not observed for the reasons described earlier. On the other hand, as shown in Figure 5d–f, the capacitive-type pressure sensor showed stable repeatability without a variation between changes in capacitance for 3000 s. However, in Figure 5a–c, slight drift and fluctuation were observed in the case of the resistive pressure sensor. Unlike the capacitive pressure sensor, the resistance-type pressure sensor did not return to its original state when the cycle was repeated and the resistance continued to decrease. This indicates that the resistance could not fully return to its initial state during the unloading process when no pressure was applied, indicating that the conductive network had not fully recovered. This phenomenon can be explained as follows. The capacitive pressure sensor measures the change in capacitance by increasing the concentration of CNTs while the composite is compressed when pressure is applied. On the other hand, the resistance-type pressure sensor measures the resistance change caused by the translational movement and deformation of the filler when pressure is applied to the composites containing the conductive filler. This can be attributed to the permanent damage to the electrically conductive network and the hysteresis effect caused by the viscoelastic matrix [49,50]. When pressure is applied, the distance between adjacent fillers decreases, forming a new conductive path, and an increase in the distance between the fillers by lateral sliding destroys the conductive path. Moreover, CNTs with high aspect ratios exhibit many complex movements [17]. Because CNTs are one-dimensional fillers, applying pressure to composites composed of CNTs can result in curvature or bending, depending on the pressure [51]. Subsequently, a change in the relative alignment may occur and the resistance may change. For this reason, a slight drift and variation were observed in the case of the resistance-type pressure sensor.

Hysteresis significantly affects sensor performance. Therefore, a one-cycle load unloading test was performed to determine the hysteresis of the sensor based on the principle of operation.

**Figure 6:**
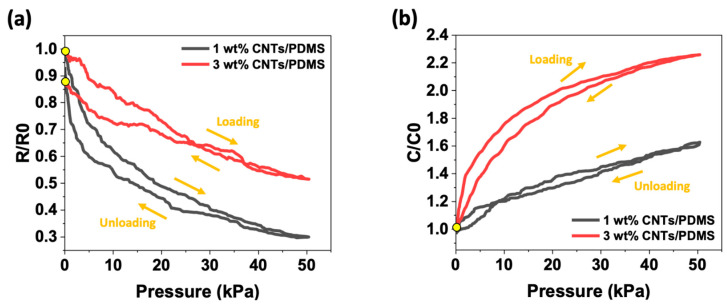
Normalized change in (**a**) resistance (*R*/*R*_0_) and (**b**) capacitance (*C*/*C*_0_) with hysteresis cycle of 50 kPa for 1 wt% and 3 wt% CNT/PDMS composites.

Figure 6 shows the hysteresis of the CNT/PDMS composites according to their working principle in the 0–50 kPa range. To compare the hysteresis of the piezoresistive and capacitive types, the 1 wt% and 3 wt% CNT/PDMS composites with the highest sensitivity were tested. In the case of the capacitive type, the 5 wt% CNT/PDMS composite showed the highest sensitivity, but 3 wt% was selected for the reasons described earlier. The curves for one cycle are shown in Figure 6a,b. Some hysteresis was observed in all composites for both properties, as shown in Figure 6. This may be due to the viscoelastic behavior of PDMS. As shown in Figure 6a, the piezoresistive type 3 wt% pressure sensor showed significantly different loading and unloading response curves, resulting in a large hysteresis. The start and end points are marked with yellow dots, but it can see that they do not overlap. In contrast, as shown in Figure 6b, the capacitive type 3 wt% pressure sensor had a relatively small hysteresis compared with the resistive type. It can be observed that the start and end points overlap. The reason for the more significant hysteresis of the resistive pressure sensor is described in the cyclic test.

As the content increased, a difference in the rate of change of resistance and capacitance according to the pressure was observed. As shown in Figure 5a, the resistivity of the 3 wt% CNT/PDMS composite was 12%, which is 10% higher than that of the 1 wt% CNT/PDMS composite. Furthermore, as shown in Figure 5b, the capacitance of the 3 wt% CNT/PDMS composite was 2% and the capacitance of the 1 wt% CNT/PDMS composite increased by 4%. Regardless of the operating principle, the hysteresis increased with increasing CNT content. This was caused by impeding the movement of more flexible polymer chains because of the more complex and interconnected fillers in the polymer [52,53]. These results indicate that a higher filler content produces greater mechanical hysteresis and that there is a correlation between the filler content and the hysteresis behavior of these nanocomposites. 

#### 3.4.3. Consideration of Temperatures

**Figure 7:**
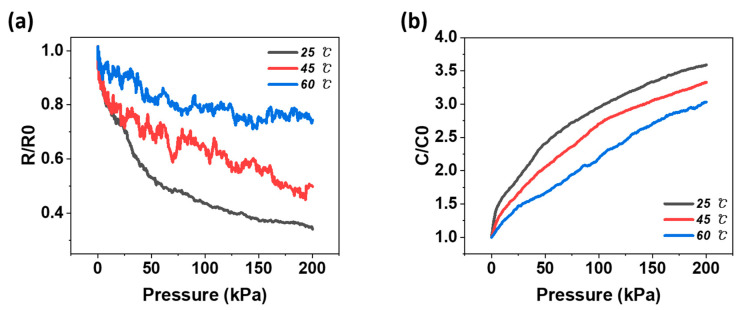
Change in (**a**) resistance (*R*/*R*_0_) and (**b**) capacitance (*C*/*C*_0_) versus pressure for 3 wt% CNT/PDMS for the reference group (25 °C, 45 °C, and 60 °C).

Sensors typically operate in highly complex environments, where the temperature varies over time. Therefore, the pressure sensor must have low-temperature dependence to maintain signal stability. In this study, three temperatures were selected as reference groups (25 °C, 45 °C, and 60 °C) to test the temperature dependence of the pressure sensor according to the working principle. The maximum temperature was selected based on the knowledge that most electrical devices do not typically exceed 65 °C. To proceed with the temperature test, a hot plate was fabricated using a CNT film and the manufactured pressure sensor was placed between and maintained at a constant temperature. A thermometer was then inserted into the composite between the electrodes to monitor whether a specific temperature had been reached. Figure 7 shows the changes in the relative resistance and relative capacitance when pressure is applied to the 3 wt% CNT/PDMS composite at the three selected temperatures.

As shown in Figure 7a, an extremely unstable change in resistance was observed in the piezoresistive-type pressure sensor. This phenomenon can be explained by the positive temperature coefficient of resistivity (PTCR) effect and the negative temperature coefficient of resistivity (NTCR) effect [54,55,56]. The PTCR effect implies that a rapid increase in resistance occurs when the temperature of some conductive composites reaches a critical point near the glass transition temperature of the polymer matrix. On the other hand, the NTCR effect refers to a phenomenon in which the resistance decreases owing to a rapid decrease in the specific resistance above a critical temperature. These effects are highly dependent on the CNT content and temperature [54]. Conductive polymer composites generally exhibit the PTCR effect, but often follow the NTCR effect when the filler content is sufficiently high. In other words, in the case of a conductive polymer composite, the dependence of the electrical resistance on temperature is a fairly complex phenomenon. The temperature coefficient of resistance can exhibit PTCR or NTCR effects, depending on the filler concentration and polymer properties. Therefore, the tunneling effect, which is the most prominent mechanism for the resistance change of the CNT/PDMS composite, and the effects described earlier can be used to explain the unstable resistance change [57]. As the temperature increased, the gap between the CNTs widened owing to the thermal expansion of the matrix, reducing the possibility of tunneling. Consequently, the temperature resistance increased, resulting in the PTCR effect. However, when the filler concentration was increased by compression, the NTCR effect appeared, resulting in an unstable resistance change. In addition, as shown in Figure 7a, the resistance change was small as the temperature increased. This is because, the higher the temperature, the greater the thermal expansion of the matrix, the wider the gap between the fillers, the more complex the tunneling effect, and the smaller the resistance change. This problem can be relieved through cyclic annealing processing. Through repeated heating and cooling, properties such as conductivity fluctuate with the cycle and eventually stabilize [58].

From Figure 7b, the relative capacitance of the pressure sensor did not change significantly as the temperature increased from 25 °C to 60 °C. The capacitance change was relatively reduced at a temperature of 60 °C, but the change was not considerable. Although the dielectric constant of the CNT/PDMS composite is affected by temperature, it is significantly smaller than its resistance [59].

As a result of all tests, the capacitive 3 wt% CNT/PDMS composite showed the highest repeatability, low hysteresis, and temperature stability under the same conditions in the basic parallel plate configuration. Therefore, it was found to be the most suitable pressure sensor for various applications.

## 4. Conclusions

CNT/PDMS composites with different filler contents were prepared to compare the pressure-sensing characteristics according to the working principle under the same conditions as the basic structure. In addition, the electrical and sensing properties of the prepared sensor samples were tested, including sensitivity, repeatability, hysteresis, and temperature dependence. First, the percolation behavior of the conductive polymer composite was measured for each working principle. It was confirmed that CNTs have a small percolation threshold owing to their high aspect ratio. As a result of the pressure test for each filler content, it was found that, the higher the filler content, the higher the pressure sensitivity of the capacitive type of composite. In contrast, for the piezoresistive type, it was confirmed that, the lower the filler content, the higher the pressure sensitivity. The main reason for this was the creation and destruction of the electrical path formation, according to the content. The 1 wt% CNT/PDMS and 3 wt% CNT/PDMS composites were selected for continuous cyclic tests according to the composition with the optimal pressure sensitivity for each working principle. Capacitive sensors exhibited more stable repeatability and hysteresis characteristics than resistance-type sensors. Finally, the temperature dependence of the 3 wt% CNT/PDMS composite was tested according to the working principle. The temperature dependence of capacitive sensors is low, so they are promising in harsh environments where temperature varies with time.

The experimental results are summarized as follows.

- The overall performance of the capacitive-type CNT/PDMS composite sensor was superior to that of the experimental piezoresistive-type sensor, including high sensitivity, good repeatability and hysteresis, and low-temperature dependence.
- It has a sensitivity of approximately 0.086 kPa^−1^ in the pressure range of 0–25 kPa and, as a result of the cyclic test in the pressure range of 0–50 kPa for 3000 s, it has excellent stability and low hysteresis of 2%.
- Piezoresistive sensors are susceptible to temperature, whereas capacitive sensors are not temperature-sensitive.

This has shown great potential for application as a pressure sensor. In conclusion, the capacitive-type CNT/PDMS composite, under the same conditions as the basic structure, is advantageous for manufacturing pressure sensors.

## Data Availability

Not applicable.
